# Acute kidney injury in a child treated with cisplatin and amphotericin B

**DOI:** 10.1007/s00280-024-04705-0

**Published:** 2024-08-07

**Authors:** Torjus Skajaa, Mia Faerch, Henrik Hasle

**Affiliations:** Department of Paediatrics and Adolescent Medicine, Aarhus University Hospital, Palle Juul-Jensens Boulevard 99, Aarhus N, 8200 Denmark

**Keywords:** Osteosarcoma, Paediatric Osteosarcoma, Nephrotoxicity, Acute kidney injury, Amphotericin B, Cisplatin

## Abstract

Cisplatin and amphotericin B are both known to be potentially nephrotoxic. We describe acute kidney injury due to the combination of Liposomal amphotericin B and cisplatin in an adolescent with osteosarcoma. Acute kidney injury (peak creatinine 431 µmol/L) consistent with drug-induced acute tubulointerstitial nephritis was observed a few days after concomitant administration of cisplatin and amphotericin B. Kidney function nearly normalised during follow-up. The timing of the concomitant administration of amphotericin B and cisplatin led us to presume that the combination was the cause of renal failure, and we conclude that concurrent administration of cisplatin and amphotericin B should be avoided.

## Introduction

Cisplatin and amphotericin B are both well known to be potentially nephrotoxic. Cisplatin can induce acute kidney injury (AKI) as a result of its toxic effects on renal tubular cells through DNA damage, oxidative stress, inflammatory responses, mitochondrial and endoplasmatic reticulum stress and impaired renal perfusion. These mechanisms collectively lead to cellular injury and death, reducing kidney function and potentially leading to acute kidney injury.

The potential nephrotoxic effect of amphotericin B is also multifactorial including direct cellular toxicity, renal vasoconstriction, and tubular damage that primarily affects the proximal tubular cells, leading to direct tubular toxicity. This results in the loss of the brush border, cellular necrosis, and tubular obstruction. An animal study has described how the combined treatment resulted in acute kidney injury [1]. However, to the best of our knowledge, no studies or case reports describe the combination of amphotericin B and cisplatin treatment in humans. Cisplatin in combination with doxorubicin and methotrexate is the cornerstone of chemo-therapy in osteosarcoma treatment. The treatment is myelosuppressive often resulting in neutropenic fever. As a result, many patients treated for osteosarcoma, will experience the situation of being treated for neutropenic fever including antifungals in relation to the treatment with chemotherapy. We outline how the combination of amphotericin B and cisplatin caused acute kidney injury in an adolescent female with osteosarcoma.

## Case report

A 17-year-old previously healthy female presented with three months of knee pain. Workup showed osteosarcoma in the right distal femur without metastases. She commenced pre-surgery chemotherapy according to the EURAMOS-1 protocol [2].

On day 27 of the first MAP series (MAP: doxorubicin 75 mg/m2, cisplatin 120 mg/m2, and two doses of high-dose methotrexate (HDM) 12 g/m2), three days prior to the second HDM course, the patient was admitted with non-neutropenic fever. First-line antibiotics with piperacillin/tazobactam 100 mg/kg three times a day were started. The patient was considered hemodynamic stable without any symptoms apart from the fever and the second dose of HDM was started according to schedule. However, the patient continued to have high temperatures during the course of chemotherapy, and on day four, antibiotics were changed to meropenem. Liposomal amphotericin B (3 mg/kg once a day) was added on day nine of fever. Chest computed tomography was suggestive of a pulmonary infection, and as the patient clinically improved (fever decreased) with the start of amphotericin B, a fungal infection was suspected. A total treatment duration of ten days of amphotericin B was administered.

The patient was treated with amphotericin B during the second dose of cisplatin (40 mg/m2, start of the second MAP series. Starting the second dose of cisplatin the patient was further treated with aprepitant (2 mg/kg) and dexamethasone (0.15 mg/kg) as anti-emetic treatment. No other nephrotoxic agents were given. Just before anti-cancer treatment commenced, and 43 days before the AKI was detected kidney function was evaluated by a diethylenetriaminepentacetate (DTPA) scan showing a normal standard glomerular filtration rate (stGFR) of 149 ml/min/1.73m2, pre-treatment plasma creatinine was 41 µmol/L, and eGFR > 90 ml/min/1.73m2 (CKD-EPI).

## Acute kidney injury

Acute kidney injury was detected three days after the second course of cisplatin. Plasma creatinine level increased to 257 µmol/L. The patient was hyperkalemic with a plasma potassium of 6.6 mmol/L and blood urea nitrogen of 15.9 mmol/L. In addition to hyperkalemia, the patient had elevated urea at 29.7 mmol/L, hyperphosphatemia at 2.5 mmol/L, and mild metabolic acidosis. Mild oliguria was treated sufficiently with IV furosemide. Hypertension reached a maximum systolic value of 151. Ultrasound of the kidneys was normal. No specific urine tests were conducted in connection with the diagnosis of the patient’s acute kidney injury (AKI), and no kidney biopsy was performed either.

Hemodialysis to clear any potentially nephrotoxic drugs from the blood was discussed but deemed without value since cisplatin is more than 90% protein-bound in the blood and therefore not dialyzable. Additionally, from a pharmacokinetic perspective, it was considered that the toxic “hit” from cisplatin had already occurred. Relevant symptomatic treatment was given for the biochemical imbalances caused by the acute kidney insufficiency. Various nephroprotective treatments were discussed, including erythropoietin (EPO) and N-acetyl cysteine (NAC), but studies at that time did not show effectiveness for these treatments.

**Fig. 1:**
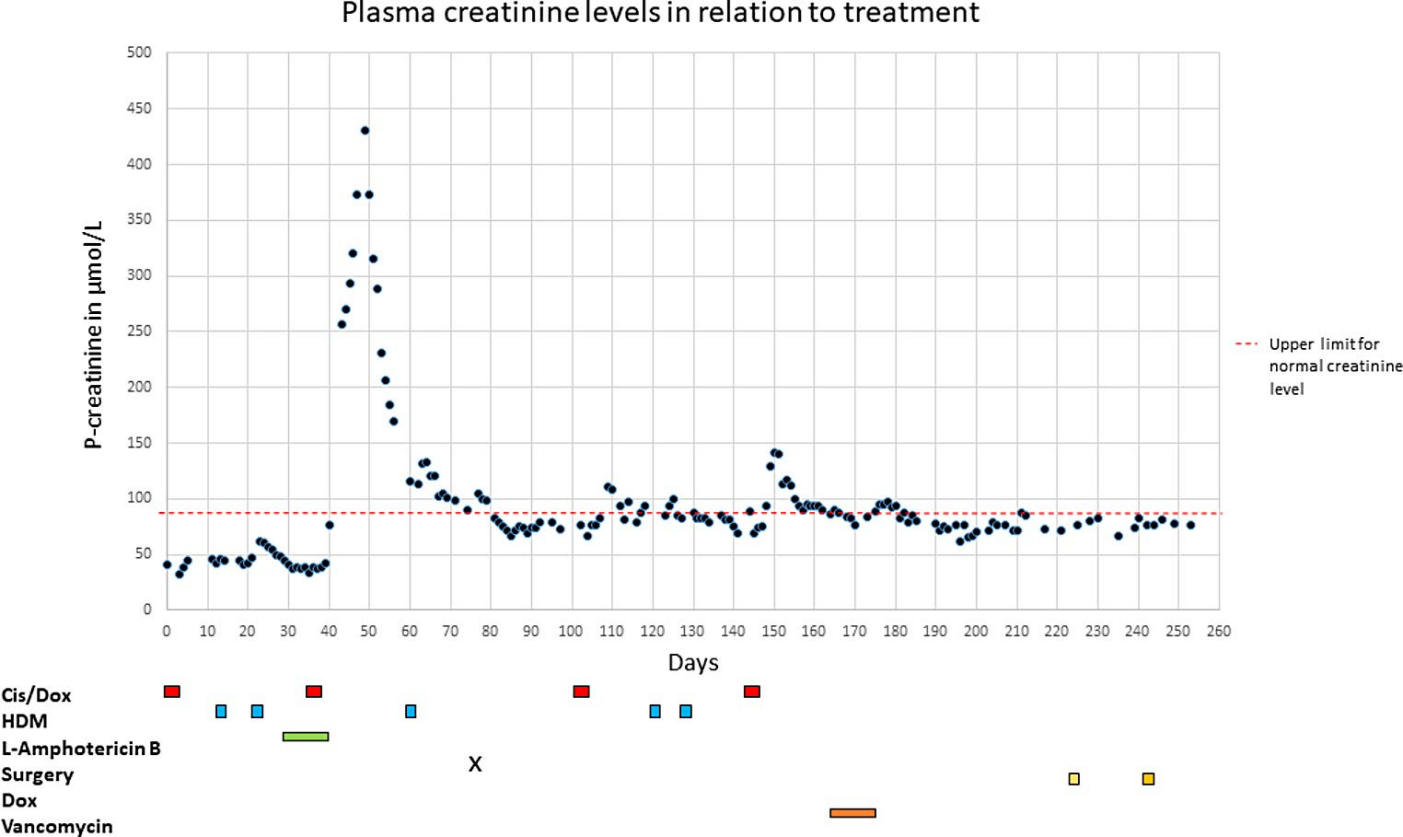
Depicting creatinine levels over time in relation to treatment. Cis/Dox: Cisplatin/Doxorubicin. HDM: High Dose Methotrexate

During the week following the initial kidney injury, plasma creatinine increased to a maximum of 431 µmol/L (Fig. 1), and a slow recovery of renal function followed, as creatinine stabilized around 100 µmol/L. Prior to the following HDM course, creatinine was 94 µmol/L, and the HDM course was given at full dose, resulting in a slight increase in creatinine that again stabilized after the course of HDM. Due to prolonged methotrexate excretion and previous kidney injury, the last pre-surgery HDM course was omitted.

In treatment week 11, as per protocol, radical limb-sparing surgery was performed, and plasma creatinine decreased further. However, we believe this decrease was more likely due to a substantial change in muscle mass following surgery than further renal recovery. This assumption was supported by a DTPA scan three months’ post-surgery showing an stGFR of 41 ml/min/1.73m2 while plasma creatinine level was 72 µmol/L and eGFR > 90 ml/min/1.73m2.

The patient completed treatment according to the Euramos-1 protocol. However, as we found 95% necrosis in the removed tumor, we opted to discard the remaining HDM courses in the last two MAP series. The patient had positive blood cultures for coagulase-negative staphylococci three months post-surgery and was treated with vancomycin (40 mg/kg/d) for 11 days. She tolerated this without any increase in creatinine. No further Amphotericin B was given throughout the treatment (Fig. 1).

### Follow-up

A DTPA scan performed ten months after the initial insult revealed an stGFR of 50 ml/min/1.73m2, with a corresponding creatinine of 89 µmol/L. Now, four years after the completion of treatment, the patient shows no signs of recurrent disease and has near-normal, stable kidney function, as determined by a creatinine level of 89 µmol/L.

## Discussion

Both amphotericin B and cisplatin are known to be potentially nephrotoxic [3–5]. An animal study has highlighted the increased nephrotoxicity when amphotericin B and cisplatin are concomitantly used [1]. However, the combination of these treatments is poorly described in humans. We present a case suggesting the potential additional effect of nephrotoxicity when both drugs are administered simultaneously. While both cisplatin and amphotericin B alone are nephrotoxic, it can be argued that each drug individually could be the sole cause of the kidney injury. We recognize that the patient didn’t receive any further liposomal amphotericin B during her treatment and thus could be the singular course of kidney injury. Liposomal amphotericin B is known to be associated with significantly less nephrotoxicity compared to conventional amphotericin B, and due to the timing of the concomitant treatment with both amphotericin B and cisplatin, as well as the minimal effect on creatinine and eGFR during the later cisplatin courses, we hypothesize the combination of amphotericin B and cisplatin was the cause of injury in this case.

As no specific urine tests nor biopsy was performed in connection with the diagnosis of the patient’s AKI it is not possible to safely say the course of injury was tubulointerstitial nephritis. However, based on the course of acute kidney impairment and the slow recovery, we believed it was highly likely consistent with drug-induced acute tubulointerstitial nephritis (ATIN). A kidney biopsy was discussed, and due to the slow improvement in kidney function along with significant neutropenia and thrombocytopenia, we refrained from it as it was not immediately considered to have any treatment consequences.

Even though plasma creatinine is now within the normal range, it is elevated as compared to pre- cancer treatment, the patient has completed puberty, regained full function of the affected limb, and recovered her muscle mass. The kidney function has been stable over the last 4 years and the patient is now into early adulthood. It is unknown when it is safe to administer cisplatin after amphotericin B, but we conclude that concurrent administration of cisplatin and amphotericin B should be avoided. Cisplatin is a key component in the anti-osteosarcoma treatment, so we suggest to select empirical anti-fungal treatment with limited nephrotoxic potential e.g. caspofungin.

## Data Availability

No datasets were generated or analysed during the current study.
